# Long-term visual outcomes and rotational stability of two diffractive toric intraocular lenses with different design combinations in cataract patients with long axial length

**DOI:** 10.3389/fmed.2026.1856827

**Published:** 2026-10-01

**Authors:** Shuang Ni, Yongwei Zhou, Zhen Zhang, Xuanzhu Chen, Limei Zhang, Haike Guo

**Affiliations:** Department of Ophthalmology, Shanghai Heping Eye Hospital, Shanghai University, Shanghai, China

**Keywords:** astigmatism, cataract, diffractive IOL, haptic design, intraocular lens, IOL material, long axial length, multifocal

## Abstract

**Background:**

Selecting intraocular lenses (IOLs) for cataract patients with long axial length remains challenging. Two diffractive toric IOLs with distinct design combinations—differing in material (hydrophobic vs. hydrophilic acrylic), optical design (trifocal vs. bifocal), haptic configuration (C-loop vs. plate-haptic), and IOL color (yellow-tinted vs. clear)—represent commonly used options. This study compared their long-term visual outcomes and rotational stability. Notably, this study was not designed to isolate individual contributions of material or IOL color, as these factors covary; posterior capsule opacification (PCO) and Nd:YAG capsulotomy rates were not systematically evaluated.

**Methods:**

This retrospective case series enrolled 57 patients (57 eyes) undergoing cataract surgery with toric multifocal IOL implantation (January–December 2023), including 29 eyes receiving the hydrophobic trifocal IOL and 28 eyes receiving the hydrophilic bifocal IOL. Sample size was based on IOL rotation as the primary endpoint (≥25 eyes per group to detect a 3° difference, 80% power, *α*=0.05). Inclusion criteria required axial length ≥25.0 mm and corneal astigmatism >0.75 D. Patients were followed at 1 week, 1 month, and 3 months postoperatively. Primary endpoint was IOL rotation at 3 months; secondary endpoints included uncorrected visual acuity, corneal astigmatism, defocus curve, objective visual quality, and satisfaction scores.

**Results:**

At 3 months, mean rotation was (2.83 ± 2.56)° in the hydrophobic trifocal group and (3.25 ± 2.29)° in the hydrophilic bifocal group (*P* > 0.05). At 1 month, uncorrected intermediate visual acuity (LogMAR) was significantly better in the hydrophobic trifocal group (0.01 ± 0.07 vs. 0.16 ± 0.09, *P* < 0.001). Cumulative uncorrected distance visual acuity of 0.1 LogMAR or better was achieved in 96.6% and 92.9% of eyes, respectively. No significant differences were found in uncorrected distance and near visual acuity, total higher-order aberrations, Strehl ratio, or questionnaire scores. Both IOLs achieved comparable visual quality and rotational stability despite design differences.

**Conclusions:**

Both diffractive toric multifocal IOLs demonstrate comparable long-term visual outcomes and rotational stability in cataract patients with long axial length. Differences in material, haptic configuration, and IOL color did not result in significant visual quality disparities. The trifocal design demonstrates advantages in intermediate visual acuity.

## Introduction

Cataract is the leading cause of blindness worldwide, and surgery remains the only effective treatment. With continuous advancements in cataract surgery techniques and increasing patient demands for visual quality, cataract surgery has evolved from a simple sight-restoring procedure to a refractive surgery. Corneal astigmatism is one of the critical factors affecting postoperative visual quality after cataract surgery. Studies have shown that approximately 80% of cataract patients in China have corneal astigmatism greater than 0.50 D, and about 41.8% have astigmatism greater than 1.00 D (1). Patients with corneal astigmatism from 1 D to 3 D account for 32.79% of cataract patients in China (2). Toric intraocular lenses (IOLs), due to their stable correction effects and strong predictability, have become one of the preferred methods for correcting corneal astigmatism in cataract patients.

In recent years, multifocal toric IOLs have been increasingly used in clinical practice, combining the dual advantages of astigmatism correction and full-range vision restoration. However, the impact of IOL material, optical design, haptic configuration, and IOL color on postoperative visual quality remains controversial, and these factors are key considerations for clinicians when selecting IOLs. It is important to note that when comparing two IOLs that differ in multiple dimensions simultaneously, it is difficult to isolate the contribution of any single factor, a limitation inherent to this type of clinical comparison.

Regarding IOL material, hydrophobic and hydrophilic acrylic materials each have their advantages and disadvantages. Traditional views suggest that hydrophobic materials have better biocompatibility and can reduce the incidence of posterior capsule opacification (PCO) (3); while hydrophilic materials, despite higher PCO rates, may reduce glistening phenomena due to their surface properties. The UK National Ophthalmology Database study showed significantly higher PCO rates with hydrophilic IOL materials compared to hydrophobic materials (4). However, Bellucci et al. (5) demonstrated that with similar optical design, there was no statistically significant difference in 5-year laser capsulotomy rates between hydrophobic and hydrophilic IOLs, suggesting that IOL posterior surface profile design may be more critical than material itself for PCO prevention. These considerations regarding material-related differences in PCO rates are relevant to long-term outcomes; however, it should be noted that the present study did not systematically evaluate PCO incidence or Nd:YAG laser capsulotomy rates, which limits the ability to draw direct conclusions about the impact of material on long-term visual outcomes.

Regarding haptic design, the rotational stability of C-loop versus plate-haptic IOLs has been a topic of academic debate. Wang et al. (6) found that plate-haptic IOLs had better stability, smaller decentration, and tilt compared to C-loop haptic IOLs. Rong et al. (7) further demonstrated that in myopic eyes, the rotation of plate-haptic toric IOLs was significantly less than that of C-loop toric IOLs, and C-loop IOL rotation was positively correlated with axial length. Zhao et al. (8) used SS-OCT dynamic observation to find that plate-haptic IOLs achieved faster capsular bag adhesion postoperatively, which may explain their better stability.

Regarding IOL color, the advantages and disadvantages of yellow-tinted (blue light-filtering) versus clear IOLs remain controversial. Yellow-tinted IOLs theoretically reduce potential retinal damage from blue light but may affect color perception. Previous studies have shown no significant differences between yellow-tinted and clear IOLs in contrast sensitivity, visual acuity, and color vision under photopic conditions, but under scotopic conditions, yellow-tinted IOLs had significantly higher color discrimination error rates in the blue light spectrum range (9). However, the present study did not include systematic color vision assessment or subjective visual questionnaires specifically evaluating color perception, which limits the ability to directly assess the impact of IOL color on visual quality.

Regarding optical design, trifocal and bifocal IOLs each have their characteristics. Trifocal IOLs can provide three focal points for distance, intermediate, and near vision, theoretically better meeting full-range vision requirements; bifocal IOLs focus on distance and near vision with relatively simpler optical design.

Previous studies have mostly focused on the impact of single factors on IOL performance, with few studies simultaneously comparing two IOLs with significant differences in material, optical design, haptic configuration, and IOL color. Two diffractive toric IOLs represent distinct technological approaches: one features hydrophobic acrylic material, trifocal optical design, C-loop haptic, and yellow-tinted optic; the other features hydrophilic acrylic material, bifocal optical design, plate-haptic, and clear optic. This “four-dimensional difference” provides a unique clinical research model, although it also means that the individual contribution of each factor cannot be isolated in this study design. Brand names are used only in the Materials and Methods section for IOL identification.

Additionally, rotational stability of toric IOLs in cataract patients with long axial length has been a clinical concern. Long axial length is associated with larger capsular bag diameter, which may theoretically increase the risk of IOL rotation (10).

Given the above background, this study was designed to compare two diffractive toric IOLs with fundamental differences in material properties, optical configuration, haptic structure, and lens color, and to analyze the impact of these factors on long-term visual function and IOL positional stability in cataract patients with long axial length, with the primary focus on comparing overall clinical performance rather than isolating individual factor contributions.

## Materials and methods

### Study population

This was a retrospective case series study. Sample size was calculated *a priori* based on the primary endpoint of IOL rotation. Using a significance level of 0.05, power of 80%, and an expected difference of 3° in rotation between groups (based on previously reported standard deviations), a minimum of 25 eyes per group was required. The enrolled sample of 29 and 28 eyes met this threshold. Fifty-seven patients (57 eyes) who underwent cataract surgery with toric multifocal IOL implantation at our hospital from January 2023 to December 2023 were enrolled. Based on the implanted IOL type, patients were divided into two groups: hydrophobic trifocal IOL group (PanOptix Toric, Alcon, USA; 29 eyes) and hydrophilic bifocal IOL group (AT LISA 909M, Carl Zeiss Meditec, Germany; 28 eyes). The mean age was 56.34 ± 15.61 years in the hydrophobic trifocal IOL group and 55.86 ± 12.62 years in the hydrophilic bifocal IOL group. Although the inclusion criteria specified age-related cataracts, the relatively young age of a subset of the cohort may reflect the inclusion of patients with early-onset cataracts associated with high myopia, which is consistent with the study population of patients with long axial length. No patients underwent refractive lens exchange. Inclusion criteria: (1) patients with implantation of the hydrophobic trifocal IOL or hydrophilic bifocal IOL for age-related cataracts; (2) axial length ≥25.0 mm; (3) preoperative corneal astigmatism >0.75 D; preoperative corneal astigmatism was further classified as with-the-rule (WTR, steep axis at 90°±30°), against-the-rule (ATR, steep axis at 0°±30°), and oblique astigmatism (steep axis at 30°−60° or 120°−150°). (4) willingness to participate in the study and complete postoperative follow-up. Exclusion criteria: (1) systemic diseases or ocular pathology (such as amblyopia, glaucoma, corneal diseases, uveitis, macular degeneration or retinopathy, neuro-ophthalmic diseases), irregular astigmatism, iris abnormalities; (2) any history of ocular surgery, ocular trauma, or ocular inflammation; (3) pathological myopia, zonular abnormalities, or capsular bag abnormalities that may lead to postoperative IOL malposition or tilt; (4) intraoperative or postoperative complications; (5) preoperative corrected distance visual acuity below 20/20; (6) inability to complete adequate follow-up. This study was approved by the hospital ethics committee, and all patients signed informed consent.

### Surgical technique

All surgeries were performed by the same experienced cataract surgeon. Preoperative biometry was performed using IOLMaster 700 (Carl Zeiss Meditec, Germany), and IOL power calculation and axis planning were performed using the Barrett Toric calculator. The target refraction was emmetropia (spherical equivalent ≤0.50 D) for all patients. The IOL constants used were A-constant 119.1 for the hydrophobic trifocal IOL and A-constant 118.5 for the hydrophilic bifocal IOL, as recommended by the manufacturers and verified against the ULIB database. A standardized surgical protocol was used in all cases, consisting of continuous curvilinear capsulorhexis, phacoemulsification, and removal of lens nucleus and cortex. The toric IOL was implanted in the capsular bag and aligned according to the preoperative planned axis. Postoperatively, antibiotic and corticosteroid eye drops were routinely used.

### Outcome measures

Primary endpoint: IOL rotation at 3 months postoperatively, defined as the absolute value of the difference between the IOL axis at the end of surgery and at 3 months postoperatively. IOL axis was evaluated using video screenshots at the end of surgery (patient in supine position) and digital slit-lamp photographs at 3 months postoperatively. Secondary endpoints: (1) uncorrected distance visual acuity (UCDVA), uncorrected intermediate visual acuity (UCIVA), uncorrected near visual acuity (UCNVA), and best-corrected distance visual acuity (BCDVA) at 1 month postoperatively; all visual acuity data are presented in LogMAR notation; cumulative visual acuity data (percentage of eyes achieving Snellen equivalents of 20/25, 20/32, and 20/40) are reported in accordance with the standards proposed by Fernández et al. (11); (2) defocus curve at 1 month postoperatively; (3) objective visual quality assessment at 1 month postoperatively, including total higher-order aberrations (HOA), coma, spherical aberration, and Strehl ratio (SR); (4) patient satisfaction questionnaire, including glare, halo, and starburst scores, and VF-14-based quality-of-life score. All secondary outcome measurements were compared between the two groups as proposed above.

### Statistical analysis

Statistical analysis was performed using SPSS 26.0 software. Continuous variables were expressed as mean ± standard deviation (mean ± SD), and between-group comparisons were performed using independent samples t-test. Categorical variables were expressed as number (percentage), and between-group comparisons were performed using chi-square test or Fisher's exact test. *P* < 0.05 was considered statistically significant.

## Results

### Baseline characteristics

Baseline characteristics of the two groups are shown in Table 1. As baseline characteristics represent the study sample (material studied), they are described here and were also considered in the Materials and Methods section. There were 29 eyes in the hydrophobic trifocal IOL group and 28 eyes in the hydrophilic bifocal IOL group. No statistically significant differences were found between the two groups in age, gender, or axial length (*P* > 0.05). Preoperative corneal astigmatism was higher in the hydrophobic trifocal IOL group than in the hydrophilic bifocal IOL group (1.79 ± 0.55 D vs. 1.46 ± 0.47 D, t = 2.44, *P* = 0.02), which is acknowledged as a limitation of this study. Preoperative corneal astigmatism was classified according to the steep axis: with-the-rule (WTR) in 8 eyes (27.6%), against-the-rule (ATR) in 20 eyes (69.0%), and oblique astigmatism in 1 eye (3.4%) in the hydrophobic trifocal IOL group, and WTR in 16 eyes (57.1%), ATR in 10 eyes (35.7%), and oblique astigmatism in 2 eyes (7.1%) in the hydrophilic bifocal IOL group (chi-square=6.32, *P* = 0.04).

**Table 1:** Comparison of baseline characteristics between the Two groups.

Parameter	Hydrophobic trifocal IOL (*n* = 29)	Hydrophilic bifocal IOL (*n* = 28)	*P* value
Age (years, mean ± SD)	56.34 ± 15.61	55.86 ± 12.62	0.90
Gender (male/female)	12/17	16/12	0.23
Axial length (mm, mean ± SD)	27.64 ± 2.06	27.82 ± 2.16	0.75
Preoperative corneal astigmatism (D, mean ± SD)	1.79 ± 0.55	1.46 ± 0.47	0.02
With-the-rule astigmatism, *n* (%)	8 (27.6%)	16 (57.1%)	—
Against-the-rule astigmatism, *n* (%)	20 (69.0%)	10 (35.7%)	—
Oblique astigmatism, *n* (%)	1 (3.4%)	2 (7.1%)	—

### Rotational stability

At 3 months postoperatively, the mean axis deviation was (2.83 ± 2.56)° in the hydrophobic trifocal IOL group (range 0°-7°) and (3.25 ± 2.29)° in the hydrophilic bifocal IOL group (range 0°−9°). The difference between groups was not statistically significant (t = −0.66, *P* = 0.52) (Figure 1). No patient had IOL rotation exceeding 10°, and no surgical repositioning was required due to rotation. The proportion of eyes with IOL rotation of 5° or less was 79.3% (23/29 eyes) in the hydrophobic trifocal IOL group and 89.3% (25/28 eyes) in the hydrophilic bifocal IOL group.

**Figure 1:**
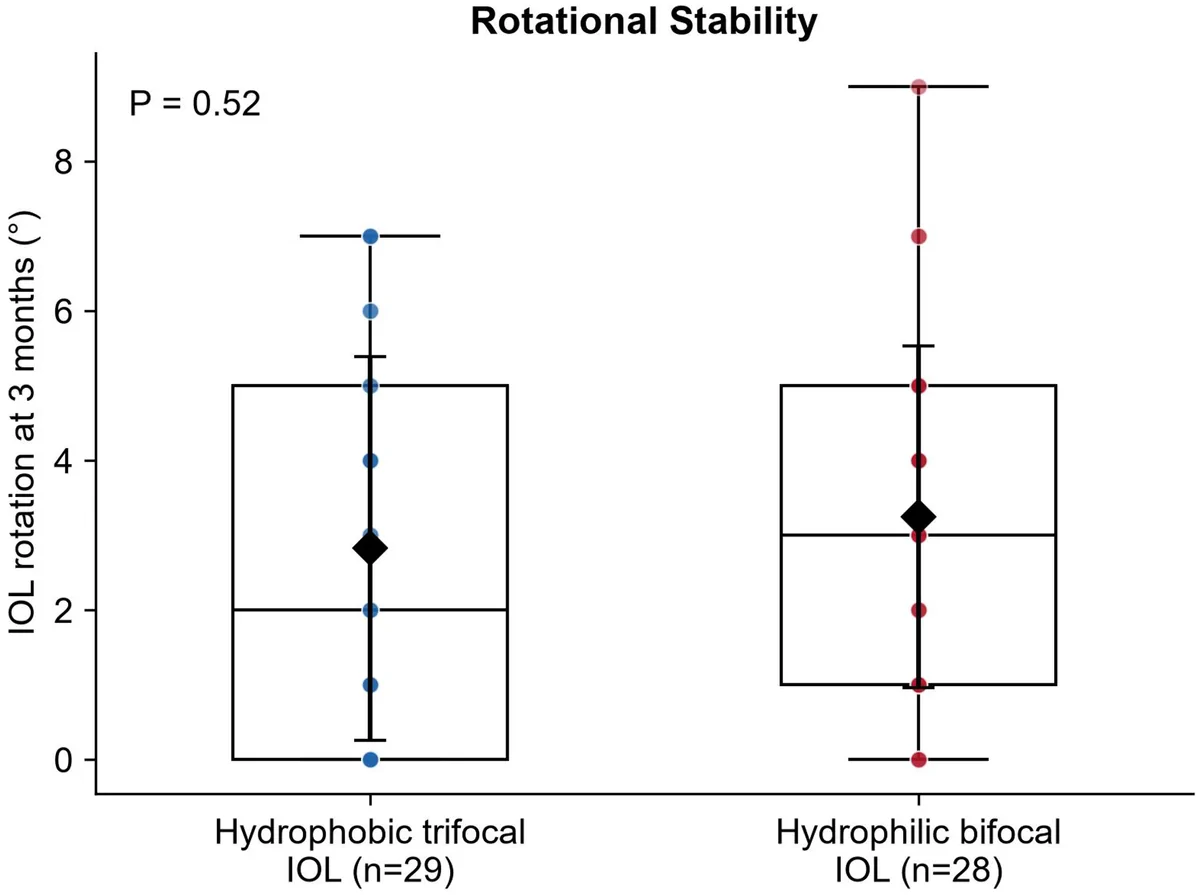
Comparison of IOL rotational stability between the two groups.

### Postoperative visual acuity

Visual acuity results at 1 month postoperatively are shown in Table 2 and Figure 2. All visual acuity values are expressed in LogMAR units. Uncorrected distance visual acuity (UCDVA) was (0.03 ± 0.06) and (0.03 ± 0.06) in the two groups, respectively, with no statistically significant difference (*P* > 0.05). Cumulative uncorrected distance visual acuity of 0.1 LogMAR (20/25) or better was achieved in 96.6% of eyes in the hydrophobic trifocal IOL group and 92.9% in the hydrophilic bifocal IOL group; 0.3 LogMAR (20/40) or better was achieved in 100% and 100%, respectively. Uncorrected intermediate visual acuity (UCIVA) was significantly better in the hydrophobic trifocal IOL group (0.01 ± 0.07) than in the hydrophilic bifocal IOL group (0.16 ± 0.09), with a statistically significant difference (t = −7.62, *P* < 0.001). Uncorrected near visual acuity (UCNVA) was (0.04 ± 0.06) and (0.06 ± 0.07) in the two groups, respectively, and best-corrected distance visual acuity (BCDVA) was (0.01 ± 0.03) and (0.02 ± 0.03), with no statistically significant differences (*P* > 0.05).

**Table 2:** Comparison of visual acuity at 1 month postoperatively (LogMAR, mean ± SD).

Visual Acuity	Hydrophobic trifocal IOL (*n* = 29)	Hydrophilic bifocal IOL (*n* = 28)	t value	*P* value
UCDVA	0.03 ± 0.06	0.03 ± 0.06	0.12	0.91
UCIVA	0.01 ± 0.07	0.16 ± 0.09	−7.62	<0.001
UCNVA	0.04 ± 0.06	0.06 ± 0.07	−1.25	0.22
BCDVA	0.01 ± 0.03	0.02 ± 0.03	−0.93	0.36

**Figure 2:**
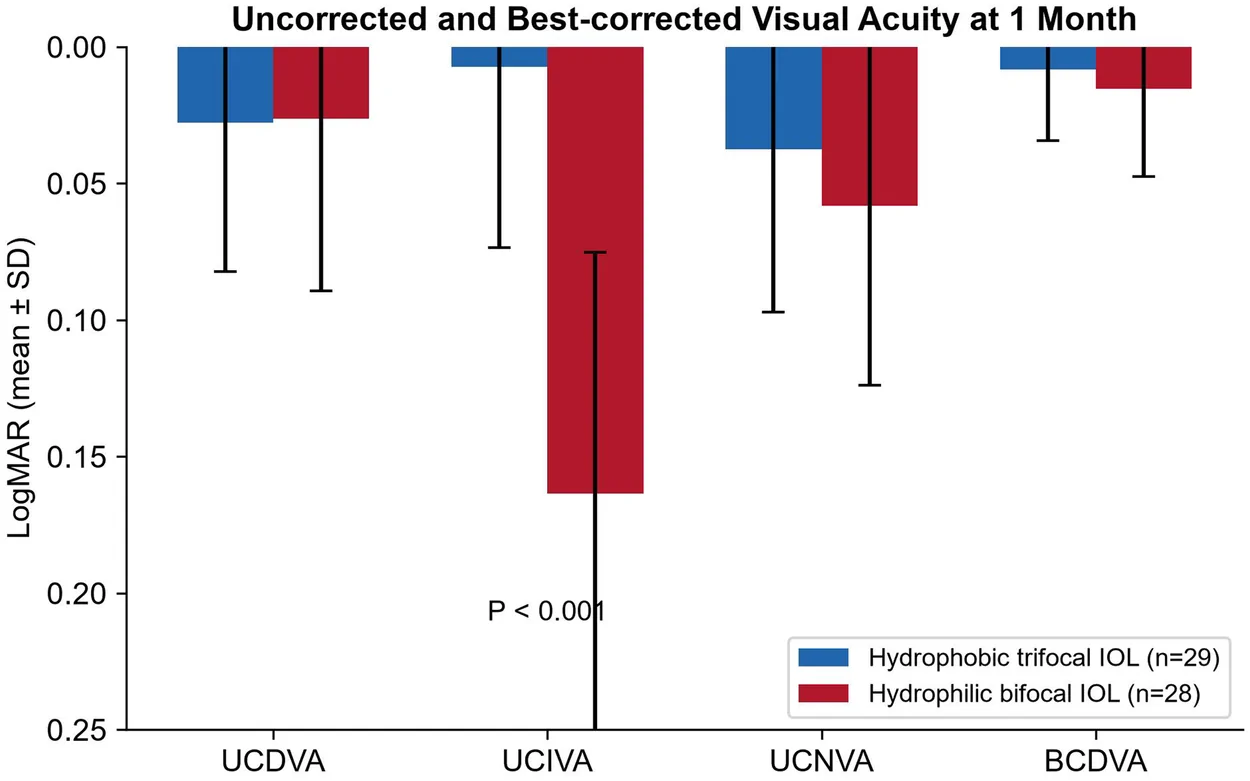
Comparison of postoperative visual acuity at 1 month between the two groups.

### Defocus curve

The defocus curves of both groups are shown in Figure 3. The hydrophobic trifocal IOL group showed visual acuity peaks at 0 D (distance), −2.0 D, and −2.5 D, reflecting the characteristics of trifocal design. The hydrophilic bifocal IOL group showed peaks at 0 D and −3.0 D, consistent with its higher near addition power. In the intermediate-to-near range (−2.0 D to −2.5 D), visual acuity was significantly better in the hydrophobic trifocal IOL group than in the hydrophilic bifocal IOL group (both *P* ≤ 0.001), whereas the hydrophilic bifocal IOL group showed better visual acuity at −3.0 D to −4.0 D and at positive defocus levels (+1.5 D to +2.0 D) (all *P* ≤ 0.004). All defocus curve values are presented in LogMAR units.

**Figure 3:**
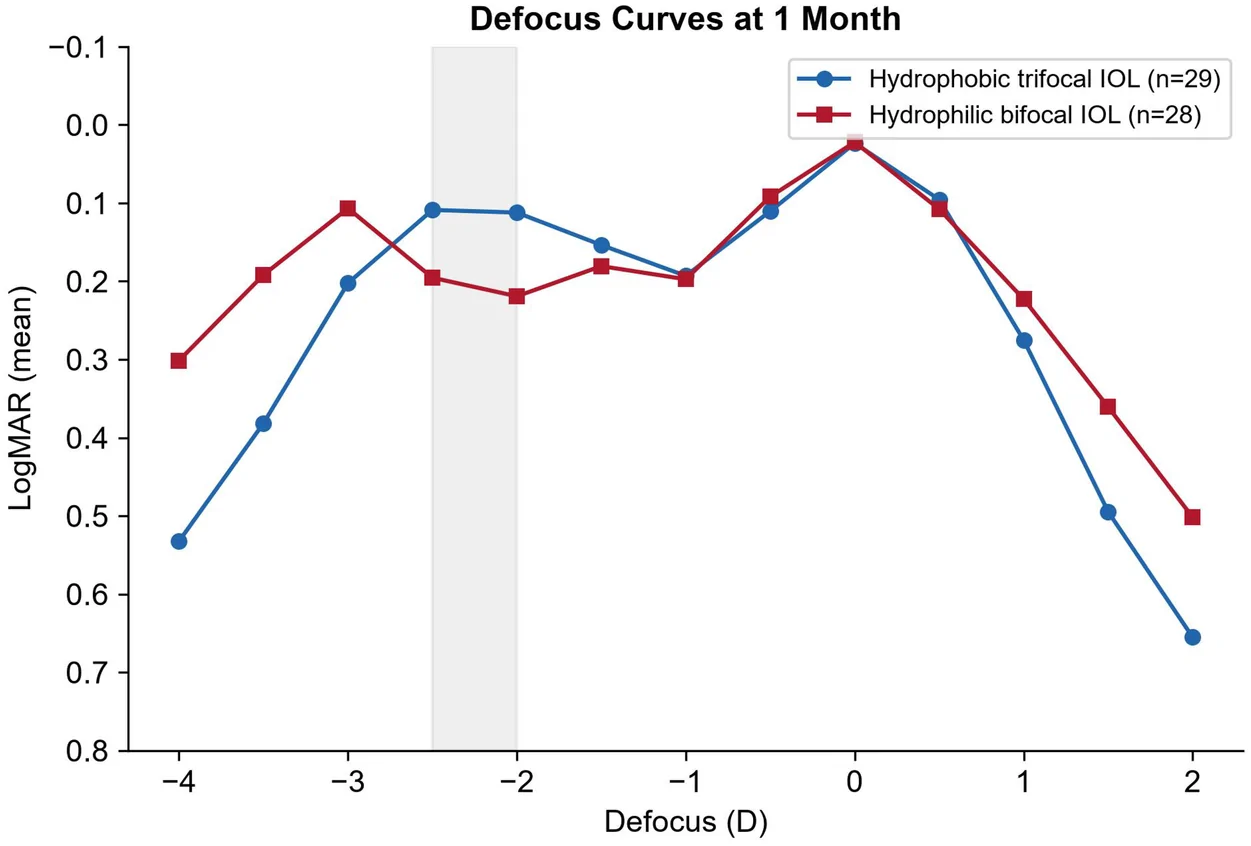
Comparison of defocus curves between the two groups.

### Corneal astigmatism

Preoperative corneal astigmatism distribution is shown in Figure 4. Preoperative corneal astigmatism was (1.79 ± 0.55) D in the hydrophobic trifocal IOL group (range 0.93–2.99 D) and (1.46 ± 0.47) D in the hydrophilic bifocal IOL group (range 0.81–2.42 D); all enrolled eyes had corneal astigmatism >0.75 D, and each eye received toric IOL implantation based on individual refractive indications. Systematic postoperative manifest refraction for vectorial analysis of residual astigmatism was not available for all eyes in this cohort and is acknowledged as a study limitation.

**Figure 4:**
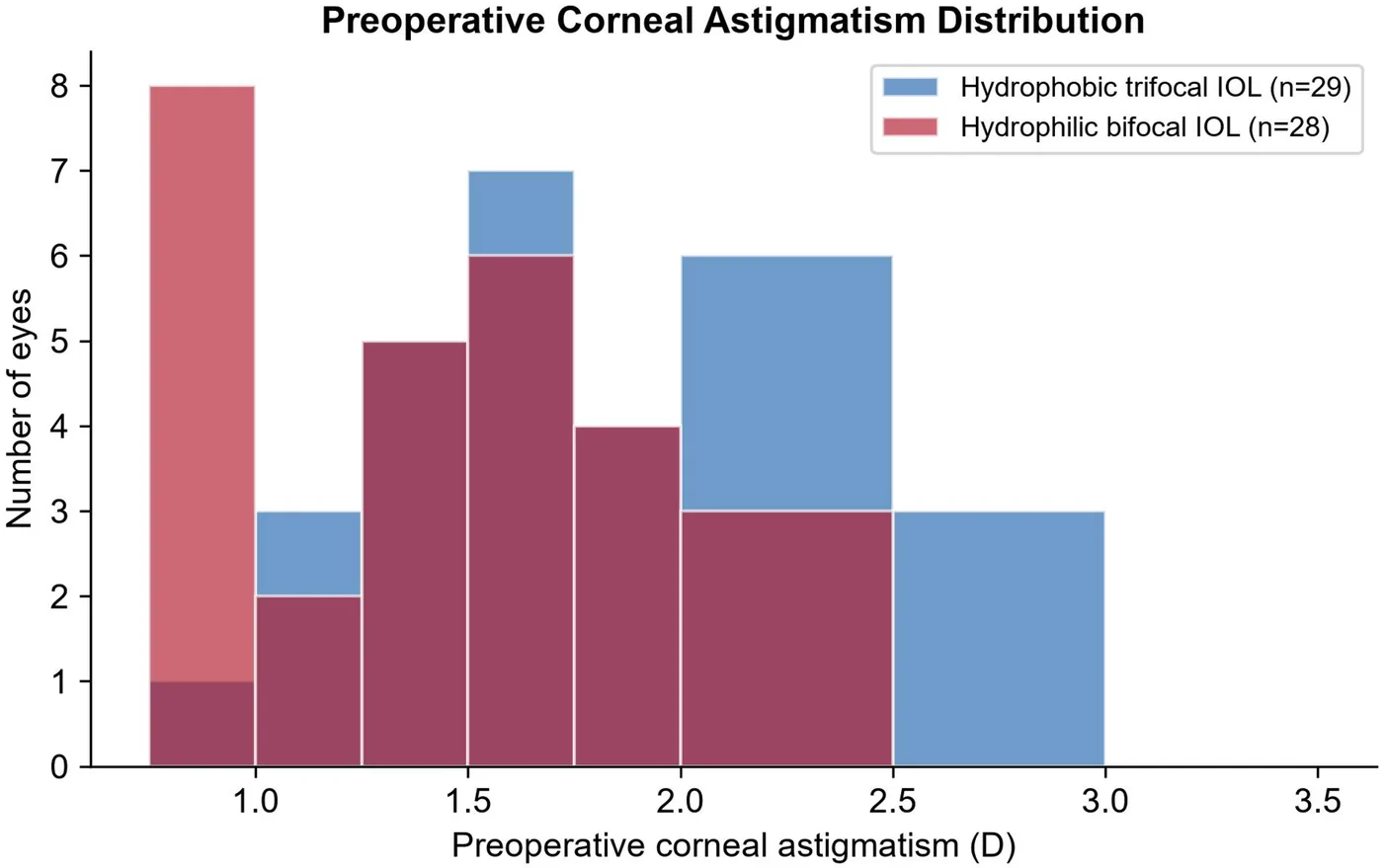
Distribution of preoperative corneal astigmatism in the two groups.

**Figure 5:**
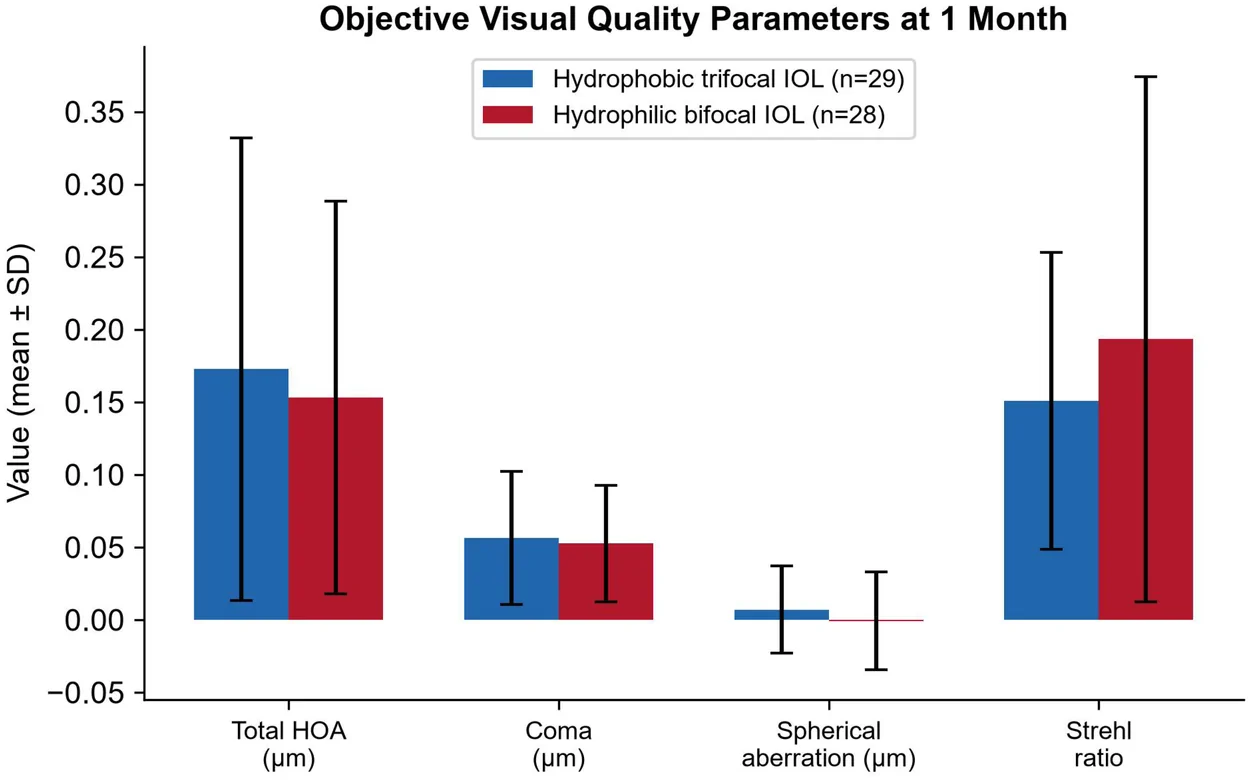
Comparison of postoperative visual quality parameters between the two groups.

### Visual quality

Postoperative objective visual quality parameters are shown in Table 3 and Figure 5. Total higher-order aberrations were (0.17 ± 0.16) μm in the hydrophobic trifocal IOL group and (0.15 ± 0.14) μm in the hydrophilic bifocal IOL group, with no statistically significant difference (*P* > 0.05). Coma was (0.06 ± 0.05) μm and (0.05 ± 0.04) μm, and spherical aberration was (0.01 ± 0.03) μm and (−0.00 ± 0.03) μm in the two groups, respectively, with no statistically significant differences (*P* > 0.05). Strehl ratio was (0.15 ± 0.10) and (0.19 ± 0.18) in the two groups, respectively, with no statistically significant difference (*P* > 0.05). Patient-reported outcomes were comparable between groups: glare (2.24 ± 1.12 vs. 2.36 ± 1.16, *P* = 0.70), halo (2.31 ± 1.07 vs. 2.36 ± 1.03, *P* = 0.87), starburst (2.48 ± 0.99 vs. 2.50 ± 1.07, *P* = 0.95), and VF-14-based quality-of-life scores (2.59 ± 0.36 vs. 2.71 ± 0.37, *P* = 0.22).

**Table 3:** Comparison of postoperative visual quality parameters (mean ± SD).

Parameter	Hydrophobic trifocal IOL (*n* = 29)	Hydrophilic bifocal IOL (*n* = 28)	*P* value
Total HOA (*μ*m)	0.17 ± 0.16	0.15 ± 0.14	0.62
Coma (μm)	0.06 ± 0.05	0.05 ± 0.04	0.75
Spherical aberration (μm)	0.01 ± 0.03	−0.00 ± 0.03	0.35
Strehl ratio	0.15 ± 0.10	0.19 ± 0.18	0.28

## Discussion

This study selected two diffractive toric IOLs with fundamental differences in material properties, optical design, haptic structure, and lens color for comparative analysis to investigate their impact on long-term visual function and rotational stability in patients with long axial length. The results present a clinically noteworthy phenomenon: despite fundamental differences in these four key dimensions, both IOLs demonstrated highly consistent long-term visual outcomes and rotational stability, with differences only in intermediate visual acuity. However, it is important to acknowledge that because these IOLs differ simultaneously in material, optical design, haptic configuration, and color, the individual contribution of each factor cannot be isolated in this study design. The following discussion therefore focuses on interpreting the observed results in the context of existing literature, while clearly delineating which findings are directly supported by our data and which represent theoretical considerations.

Regarding the impact of IOL material on long-term visual quality, traditional views suggest that hydrophobic acrylic materials have better biocompatibility and can reduce PCO incidence. The mechanism involves stronger adhesion between hydrophobic materials and the posterior capsule, preventing lens epithelial cell migration to the posterior capsule (3). Conversely, hydrophilic materials are thought to have higher PCO rates and may affect IOL positional stability due to capsular bag shrinkage. However, this study showed no statistically significant differences in visual quality parameters between the hydrophobic trifocal IOL group and the hydrophilic bifocal IOL group, consistent with Bellucci et al. (5). Bellucci et al. conducted a 5-year follow-up of hydrophobic and hydrophilic IOLs with similar optical designs and found no significant differences in refractive outcomes, aberrations, contrast sensitivity, or laser capsulotomy rates. This finding suggests that IOL posterior surface profile design (especially sharp edge design) may be more critical than material itself for PCO prevention. Sharp edges can form a mechanical barrier within the capsular bag, preventing lens epithelial cell migration, and this effect may have similar mechanisms across different materials. It must be emphasized, however, that this study did not systematically evaluate PCO incidence or Nd:YAG laser capsulotomy rates. Therefore, while the visual quality parameters assessed were comparable between groups, the long-term impact of material on PCO development and subsequent visual deterioration cannot be directly confirmed by our data. This is a significant limitation, as PCO and YAG capsulotomy rates are particularly relevant to long-term follow-up outcomes, especially for visual acuity at 1 or 2 years postoperatively. Future studies incorporating systematic PCO assessment and YAG rate documentation are needed to draw definitive conclusions regarding the impact of IOL material on long-term outcomes.

Regarding the impact of haptic design on rotational stability, previous study results have been inconsistent. Wang et al. (6) found that plate-haptic IOLs had better stability, smaller decentration, and tilt compared to C-loop haptic IOLs. Rong et al. (7) further confirmed that in myopic eyes, the rotation of plate-haptic toric IOLs (4.42 ± 3.24)° was significantly less than that of C-loop toric IOLs (8.00 ± 3.60)°, and C-loop IOL rotation was positively correlated with axial length, while plate-haptic IOLs showed no such correlation. Zhao et al. (8) used SS-OCT dynamic observation to find that plate-haptic IOLs achieved faster capsular bag adhesion in the early postoperative period, which may explain their better stability. However, this study showed no statistically significant difference in rotational stability between the C-loop hydrophobic trifocal IOL group and the plate-haptic hydrophilic bifocal IOL group ((2.83 ± 2.56)° vs. (3.25 ± 2.29)°, *P* = 0.52). This result may be related to several factors: first, optimization of modern C-loop designs may have significantly improved their stability; second, the capsular bag morphology in patients with long axial length may make haptic impact less significant.

Regarding the impact of IOL color on visual quality, the controversy between yellow-tinted (blue light-filtering) and clear IOLs has a long history. Yellow-tinted IOLs filter short-wave blue light in the 400–500 nm range, theoretically reducing retinal light damage risk, but their impact on color vision and contrast sensitivity has been a concern. Mueller et al. (9) showed that yellow-tinted and clear IOLs had no statistically significant differences in contrast sensitivity, visual acuity, and color vision under photopic conditions, but under scotopic conditions, yellow-tinted IOLs had significantly higher color discrimination error rates in the blue light spectrum range. This study showed no statistically significant difference in contrast sensitivity-related parameters (such as Strehl ratio) between the yellow-tinted hydrophobic trifocal IOL group and the clear hydrophilic bifocal IOL group, consistent with previous studies. Notably, the hydrophobic trifocal IOL group had slightly lower glare scores than the hydrophilic bifocal IOL group, although not statistically significant, suggesting yellow-tinted IOLs may have some advantage in reducing glare. A previous comparative study (12) further confirmed no significant differences in color vision parameters between yellow-tinted and clear IOLs, with differences only in scatter index, suggesting the impact of blue light-filtering function on color vision may be overestimated. However, it must be clearly stated that this study did not include systematic color vision assessment or subjective visual questionnaires specifically evaluating color perception. Therefore, while our objective visual quality parameters showed no significant differences between the yellow-tinted and clear IOL groups, the impact of IOL color on color vision and subjective visual experience cannot be directly assessed based on our data. The discussion of color-related differences in the existing literature is provided for context, but should not be interpreted as findings supported by the present study.

In terms of optical design, the difference between trifocal and bifocal IOLs was fully demonstrated in this study. The hydrophobic trifocal IOL group showed significantly better intermediate visual acuity than the hydrophilic bifocal IOL group, consistent with the design characteristics of trifocal IOLs. The hydrophobic trifocal IOL uses ENLIGHTEN optical technology with a near addition power of +3.25 D (at the IOL plane) and an optimal intermediate focal point at 60 cm, better suiting Asian visual habits (13). The hydrophilic bifocal IOL has a near addition power of +3.75 D (at the IOL plane) with a near focal point at approximately 40 cm. The difference in near addition power (+3.25 D vs. + 3.75 D) may influence the near visual acuity outcomes; however, our results showed no statistically significant difference in UCNVA between the two groups, which may be related to the measurement distance of near visual acuity at 40 cm in this study. It is worth noting that the near addition power at the IOL plane translates to different effective additions at the corneal plane depending on axial length; in eyes with longer axial length, the effective near addition may be slightly reduced, which could influence near visual acuity and the shape of the defocus curve. Defocus curve analysis further confirmed the intermediate visual acuity difference, with better visual acuity in the hydrophobic trifocal IOL group in the −2.0 D to −2.5 D range, whereas the hydrophilic bifocal IOL group performed better at nearer distances (−3.0 D to −4.0 D), consistent with the difference in near addition power between the two IOLs. Conversely, the better visual acuity of the hydrophilic bifocal IOL group at positive defocus levels (+1.5 D to +2.0 D) may be explained by the diffractive light-energy distribution of the two designs: the allocation of light energy to the intermediate focus in trifocal IOLs may reduce defocus tolerance on the distance side, whereas bifocal designs concentrate their diffractive energy on the distance and near foci. Additionally, the influence of axial length on defocus curve results should be considered: longer eyes may exhibit different effective defocus profiles due to the altered relationship between IOL power and axial length. This finding has guiding significance for clinical IOL selection: for patients with intermediate visual needs, trifocal IOLs may be the better choice.

This study has certain limitations. First, the sample size was relatively small, which may affect statistical power, especially for the reliability of subgroup analyses. Second, as a retrospective study, there may be selection bias. Third, there was a statistically significant difference in preoperative corneal astigmatism between the two groups, which may partly reflect indication-based IOL allocation and residual confounding cannot be excluded. Fourth, this study did not systematically evaluate PCO incidence or Nd:YAG laser capsulotomy rates, preventing direct verification of material impact on PCO and long-term visual outcomes. Fifth, systematic postoperative manifest refraction for residual astigmatism analysis was not available for all eyes in this cohort. Sixth, this study did not include systematic color vision assessment, limiting the ability to evaluate the impact of IOL color on visual quality. Seventh, as the two IOLs differ simultaneously in material, optical design, haptic configuration, and color, the individual contribution of each factor cannot be isolated in this study design. Future larger sample size, prospective randomized controlled studies with PCO assessment are needed for further verification.

In conclusion, both diffractive toric multifocal IOLs, despite significant differences in material, optical design, haptic configuration, and IOL color, provide satisfactory long-term visual outcomes and rotational stability in cataract patients with long axial length (AL ≥25.0 mm). While conventional wisdom suggests potential limitations associated with certain material properties, C-loop haptic design, and yellow-tinted optics, this study did not find significant visual quality disparities between the two IOL designs. The trifocal IOL demonstrates advantages in intermediate visual acuity.

## Data Availability

The raw data supporting the conclusions of this article will be made available by the authors, without undue reservation.
